# Precipitation pattern changed the content of non-structural carbohydrates components in different organs of *Artemisia ordosica*

**DOI:** 10.1186/s12870-023-04512-4

**Published:** 2023-10-21

**Authors:** Yingying He, Minghan Yu, Guodong Ding, Fuchong Zhang

**Affiliations:** 1 College of Forestry and Prataculture, Ningxia University, Yinchuan, 750021 China; 2 Yanchi Research Station, School of Soil and Water Conservation, Beijing Forestry University, Beijing, 100083 China; 3 Key Laboratory of State Forestry Administration on Soil and Water Conservation, Beijing Forestry University, Beijing, 100083 China

**Keywords:** *Artemisia ordosica*, Desert plants, Non-structural carbohydrate, Precipitation, Precipitation interval

## Abstract

**Background:**

Non-structural carbohydrates (NSC) play a significant role in plant growth and defense and are an important component of carbon cycling in desert ecosystems. However, regarding global change scenarios, it remains unclear how NSCs in desert plants respond to changing precipitation patterns. [Methods] Three precipitation levels (natural precipitation, a 30% reduction in precipitation, and a 30% increase in precipitation) and two precipitation intervals levels (5 and 15 d) were simulated to study NSC (soluble sugar and starch) responses in the dominant shrub *Artemisia ordosica*.

**Results:**

Precipitation level and interval interact to affect the NSC (both soluble sugar and starch components) content of *A. ordosica*. The effect of precipitation on NSC content and its components depended on extended precipitation interval. With lower precipitation and extended interval, soluble sugar content in roots increased and starch content decreased, indicating that *A. ordosica* adapts to external environmental changes by hydrolyzing root starch into soluble sugars. At 5 d interval, lower precipitation increased the NSC content of stems and especially roots.

**Conclusions:**

*A. ordosica* follows the “preferential allocation principle” to preferentially transport NSC to growing organs, which is an adaptive strategy to maintain a healthy physiological metabolism under drought conditions. The findings help understand the adaptation and survival mechanisms of desert vegetation under the changing precipitation patterns and are important in exploring the impact of carbon cycling in desert systems under global environmental change.

**Supplementary Information:**

The online version contains supplementary material available at 10.1186/s12870-023-04512-4.

## Background

Water is an important environmental limiting factor for plant growth that significantly impacts plant survival, growth, and distribution, particularly in nutrient- and water-limited desert ecosystems [1, 2]. In the context of climate change, global warming will alter global and regional water cycles, resulting in future changes in local precipitation patterns, including precipitation amount and frequency [3, 4]. Extreme precipitation events associated with climate change and greater interannual precipitation variability have become significant factors affecting regional vegetation stability in the sandy areas of Northwest China [5].

The regulation of non-structural carbohydrate (NSC) allocation is a central plant response strategy to precipitation changes. NSCs are important for plant metabolism and the main storage form of energy [6, 7]. Soluble sugars and starches are the main components of NSC and account for more than 90% of all NSC in plants [8]. As the main form of NSC transport in plants, soluble sugars regulate osmotic pressure and maintain metabolic functions [9]. Starch is an important energy-storage substance in plants [10]. Several studies have shown that during the dry season, plants break down starch into soluble sugars to maintain constant the total NSC content and thereby preserve normal physiological activity, suggesting that precipitation affects the proportion of different NSC types in plants [11, 12]. In addition, in response to water scarcity, some plants transfer and store large amounts of NSCs in the root system and increase the energy storage allocation in the root system in order to improve water acquisition opportunities [13, 14]. Therefore, the allocation of NSC regulation among different organs is also an adaptive strategy for plants to cope with changes in water availability [15, 16]. Studying the dynamics of NSCs and their response to environmental conditions, particularly rainfall, is important for understanding plant adaptation mechanisms. it also provides a basis for assessing organ, individual, and ecosystem carbon balances, as well as ecosystem succession [17, 18]. Previous studies have mostly focused on NSC changes and component characteristics of individual or partial organs [19]. however, studies on synergistic changes in NSCs across all organs of a plant are lacking.

Numerous studies have examined how NSC content responds to precipitation changes. Some studies have suggested that precipitation reduction can increase plant NSC content [20, 21], while others show that precipitation reduction can decrease plant NSC content [7, 22]. In contrast, some plant species can maintain stable NSC content under different precipitation conditions by adjusting the ratio of soluble sugars to starch [23–25]. Consequently, there is no uniform understanding of the impact of precipitation variation on plant NSC content. The response of plant NSC to precipitation exhibits greater variability and complexity due to the diverse characteristics of precipitation allocation, including frequency and duration [26]. Studies have shown that plant NSC content may increase in the early stages of drought [27, 28], but with the continuation of drought, plant NSC, including soluble sugar and starch, content decreases [26]. Although there are abundant studies on the influence of precipitation on plant NSC content, the influence of precipitation duration remains poorly understood [29–31], and the interaction between the two on plant NSC content is rarely discussed, which greatly restricts the understanding and prediction of changes in plant NSCs and their constituents.

*Artemisia ordosica* is a dominant and constructive species that covers 31% of the Mu Us Sandy Land of Northwestern China and plays an important role in the stability of the ecosystem in the region. Therefore, in this study, taking *A. ordosica* as the research object, we conducted a field control experiment simulating three levels of precipitation (W-30%, W, W + 30%) and two levels of precipitation intervals (T, T++) in a desert shrubland in the Mu Us Desert of northern China and examined the effects of changes in precipitation patterns on NSC storage at the whole-plant and organ levels. The study addresses the following three question: (1) Will the alteration in precipitation patterns have an effect on the NSC, starch and soluble sugar content of *A. ordosica*? (2) Do soluble sugars and starch transform each other to adapt to precipitation change? (3) Does the distribution ratio of plant NSC among different organs change due to precipitation change?

## Materials and methods

### Study site

The experimental area was at the Yanchi Research Station in Ningxia City on the southwest edge of Mu Us Sandy Land, China (37°42’31"N, 107°13’37"E, 1530 m above sea level). The area has a typical temperate continental monsoon climate with average annual precipitation and evaporation of 280 and 2024 mm, respectively. Precipitation is mainly concentrated in summer and autumn, with 83.3% of annual precipitation occurring from May to September. Annual average temperature is 8.1 ℃, annual sunshine duration is approximately 2867.3 h, and the frost-free period is approximately 165 d. The prevailing wind is northwesterly, with average annual wind speed of 2.8 m/s, and mainly concentrated from November to April of the following year. The experimental site has a loose soil structure and is dominated by aeolian sandy soils. The main plant species in this region are *A. ordosica*, *Hedysarum mongolicum*, *Salix psammophila*, *Leymus secalinus*, *Heteropappus altaicus*, *Pennisetum centrasiaticum*, *Corispermum puberulum*, *Setaria viridis*, and *Cynanchum thesioides*. We chose *A. ordosica* as our study species. It is a deciduous dwarf shrub, the average height of mature plants is between 50 and 100 cm, and the branches are mainly yellowish brown or blackish grey. Normally we divide the branches into vegetative twigs and reproductive twigs by the maturity of their growth. In semi-fixed and fixed sands, the root system of *A. ordosica* can usually penetrate the soil layer mainly 20–45 cm deep, and in mobile sands, the root system of *A. ordosica* penetrates 100–200 cm, and can keep the normal growth of the plant by absorbing the deep soil groundwater. *A. ordosica* is an excellent sand-fixing half-shrub plant endemic to arid and semi-arid desert areas in China, widely distributed in fixed and semi-fixed sandy areas in Inner Mongolia, Ningxia, Shaanxi, Gansu, and other provinces, exhibiting characteristics such as resistance to wind erosion and barrenness, and playing an important role in ecological construction of sandy area vegetation.

### Cultivation of experimental material

The experiment was conducted from March to October in 2020 and 2021 at the Yanchi Research Station in Ningxia City. In late March 2021, the sandy soil in the study area was screened as cultivation soil, loaded into PVC buckets (height 60 cm, diameter 26 cm), and buried in the soil to prevent the influence of soil moisture on the experimental treatment. In addition, 10 *A. ordosica* seeds were sown in each PVC bucket. After the plants grew, any excess *A. ordosica* were removed at seedling stage, leaving only one per bucket. The height of the PVC buckets was sufficient for *A. ordosica* roots to grow, as the roots of *A. ordosica* are mainly distributed between 0 and 50 cm depth. After two months of slow growth, precipitation treatment started in early June.

### Experimental design

The experiment used a two-factor (precipitation amount and precipitation interval) level combination, and the precipitation amount treatment set the precipitation gradient according to the average monthly precipitation amount during the regional growing season (May–October). Regional precipitation statistics show that average annual precipitation amount from 1990 to 2017 was 311 mm, varying by approximately 30% around this annual average (highest, 449 mm; lowest, 212 mm). The experiment therefore tested three corresponding precipitation levels: annual mean natural precipitation amount (W), W decreased by 30% (W-), and W increased by 30% (W+). The annual precipitation data (1990–2017) show that precipitation frequency is generally stable within the study area, with the largest proportion of precipitation events (62.09%) occurring within a time interval of 0–5 d. Precipitation intervals in the arid and semi-arid regions of Northwest China are expected to increase in the future. Therefore, in this experiment, a precipitation interval of 5 d was selected to simulate the natural precipitation frequency (T). To account for the increased precipitation levels associated with prolonged rainfall events, this interval was extended to 15 d to simulate periods of heavy rainfall (T++). The experiment involved six treatment levels: W-T, WT, W + T, W-T++, WT++, and W + T++. The WT level represents the treatment with natural precipitation amount and precipitation frequency and served as the control group in this experimental series. Our experiment used the randomized complete block design with 3 replicates per treatment. The gradients of total monthly precipitation amount and precipitation interval were controlled by artificial irrigation following the experiment design.

### Field sampling and measurements

During sampling in late October, the PVC buckets were dug out of the soil. The whole *A. ordosica* plants were removed from the buckets to ensure the plants are intact. Each plant was then divided into three parts: leaves, stems, and roots, placed in an oven at 120 °C for 30 min, and then dried at 70 °C to a constant weight. After crushing, the material was passed through a 0.4 mm sieve and stored prior to determining the starch and soluble sugar contents. Soluble sugar and starch contents were determined from the absorbance at 625 nm of the same anthrone reagent in a spectrophotometer. The soluble sugar content was based on the regression equation of a standard solution of glucose, while the glucose content multiplied by a conversion factor of 0.9 was calculated as the starch content. Soluble sugar and starch contents were shown as a percentage of dry matter [22, 31]. In this experiment, the NSC content was calculated as the sum of soluble sugars and starch. The soluble sugar, starch, and NSC contents of the whole *A. ordosica* plants were calculated following Eq. (1):

1 $${\varvec{C}}_{Plant}=\frac{\sum {\varvec{C}}_{Organ}\times {\varvec{B}}_{Organ}}{{\varvec{C}}_{Organ}}$$

where *C*_*Plant*_ and *C*_*Organ*_ represent the content (%) of NSCs and their carbon components (soluble sugar and starch) in the whole plant and different organs of *A. ordosica*, respectively. *B*_*Organ*_ represents the biomass (g) of each organ of *A. ordosica.*

### Statistical analyses

Microsoft Excel 2019 was used to input and collate the experimental data, and SPSS software (version 26.0; IBM Corp., Armonk, NY, USA) was used for statistical analysis. Two-way ANOVA was used to test the differences in the contents of NSCs and their components in whole *A. ordosica* plants by precipitation amount and precipitation intervals. One-way ANOVA was used to test for differences in the contents of NSCs and their components among different precipitation amounts, precipitation intervals, and plant organs. Duncan’s test was used to make multiple comparisons. Origin 2022b data analysis and graphing software (OriginLab, Northampton, MA, USA) was used to provide graphical representations of the data.

## Results

### Effects of precipitation amounts and interval on NSC in *A. ordosica*

**Fig. 1:**
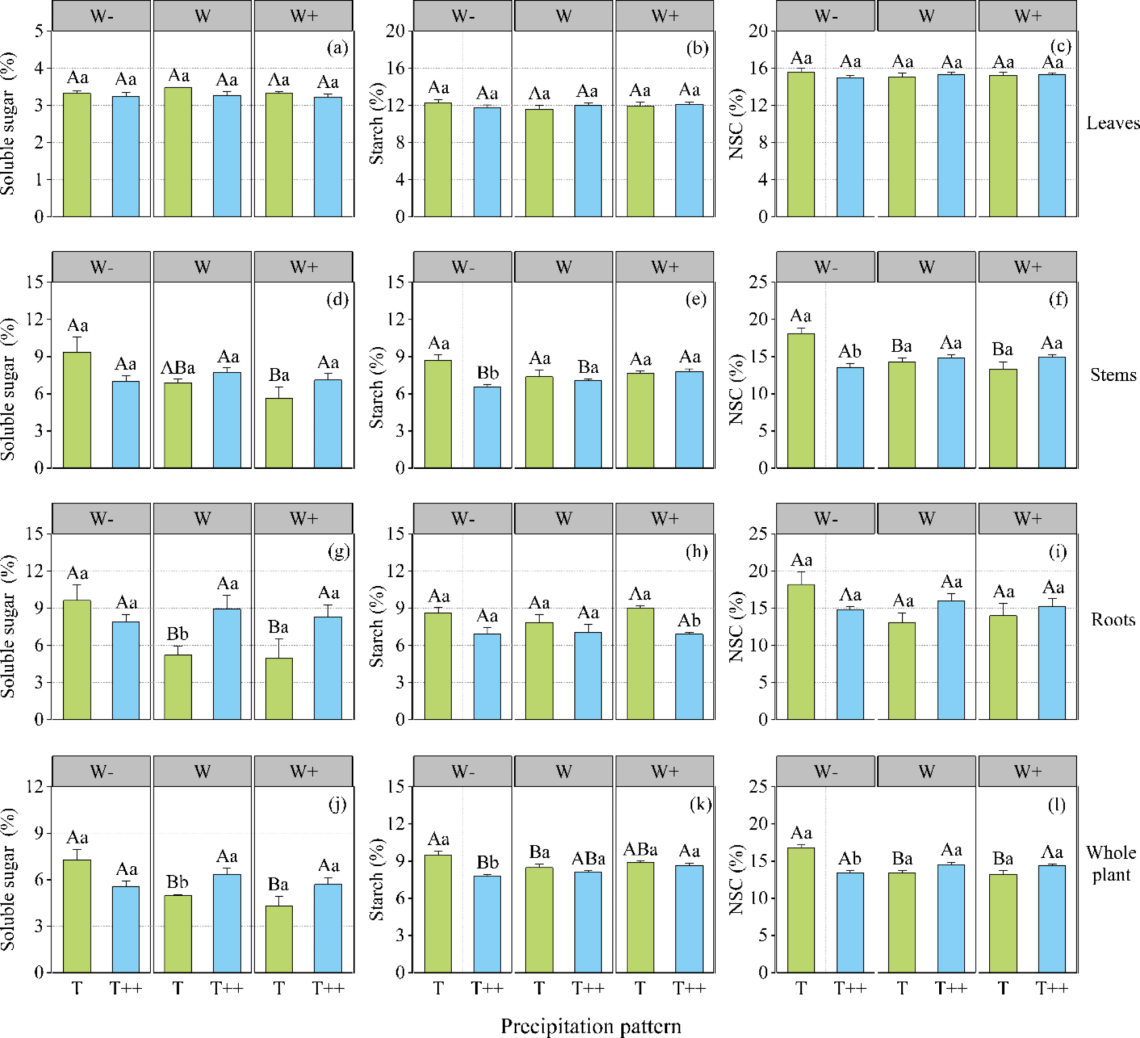
Effects of changes in precipitation pattern on non-structural carbohydrates (NSCs) and their components in *Artemisia ordosica*. Soluble sugar (**a, d, g, f**), starch (**b, e, h, k**), and the total NSC pools (**c, f, i, l**) at the organ and whole plant levels in *A. ordosica* seedlings. Error bars indicate ± 1 S.E. (*N* = 3). Note: Data in the figure are presented as the mean ± standard error (*n* = 3). Different uppercase letters show significant differences in precipitation amount under the same precipitation interval (P < 0.05); different lowercase letters indicate significant differences between the different precipitation intervals at the same precipitation level (P < 0.05)

As shown in Fig. 1, at the T level, the soluble sugar content of *A. ordosica* in stems and roots followed the sequence: W- > W > W+. The content of NSCs in stems was as follows: W- > W > W+, whereas the contents of soluble sugar and NSCs in leaves and the content of NSCs in roots did not show significant differences among different precipitation amount treatments. At the level of T++, the contents of soluble sugar and NSCs in each organ showed non-difference under the different precipitation amount treatments, and the content of starch in stems was as follows: W- < W < W+. In other words, at natural precipitation frequency, the decrease in precipitation amount increased soluble sugar content of stems and total NSCs. However, at longer precipitation intervals, the decrease in precipitation amount increased the starch content of stems.

At the W- level, the soluble sugar content of each organ of *A. ordosica* was not affected by the precipitation interval, and the contents of starch and NSCs in stems were in the order of T > T++; the starch and NSC contents of the leaves and roots were not affected by the precipitation interval. At the W level, the starch and NSC contents in each organ showed non-difference under different precipitation intervals, and the soluble sugar content in the roots of *A. ordosica* was lower at T than at T++. At the W + level, the contents of soluble sugar and NSCs in each organ of *A. ordosica* were not affected by the precipitation interval, and the starch content in roots was higher at T than at T++. In conclusion, under the background of natural precipitation amount, extended precipitation intervals increased the soluble sugar content of the roots of *A. ordosica*. With 30% reduction in the precipitation amount, longer precipitation interval was associated with lower starch content in stems of *A. ordosica*. However, when the precipitation amount increased by 30%, longer precipitation interval decreased the starch content in the root system.

As shown in Table 1, the precipitation interval had a significant effect on the starch content of *A. ordosica* stems and roots (P < 0.05), whereas precipitation amount had no significant effect on the content of NSCs or their components in each organ. The interaction between precipitation amount and precipitation interval had significant effects on the total content of soluble sugar, starch, and NSCs in the stems of *A. ordosica* (P < 0.05), and had a significant effect on the soluble sugar content of roots (P < 0.05).

**Table 1:** Significance of multivariate analysis of variance for non-structural carbohydrate (NSC) content with precipitation amount, precipitation interval, and organs (*F*-value)

Factor	Organ	Soluble sugar	Starch	NSC
W	Leaves	0.960	0.197	0.051
	Stems	3.128	1.309	3.499
	Roots	2.146	0.565	1.545
	Whole plant	4.704*	2.866	7.114**
T	Leaves	3.876	0.022	0.096
	Stems	0.002	8.079*	2.116
	Roots	3.990	14.681**	0.047
	Whole plant	0.583	20.707***	2.424
W×T	Leaves	0.376	1.069	0.917
	Stems	4.063*	6.675*	12.095**
	Roots	3.978*	0.901	3.278
	Whole plant	7.103**	7.904**	23.831***

Note. ∗: P < 0.05, ∗∗: P < 0.01, ∗∗∗: P < 0.001. W: precipitation amount; T: precipitation interval

It can be seen from Fig. 1j, k, l that at the T level, the contents of soluble sugar, starch, and NSCs in whole *A. ordosica* plants followed the sequence: W- > W > W+, that is, the soluble sugar, starch, and NSC contents of the whole *A. ordosica* plant increase when precipitation decreases. At the level of T++, the starch content of the whole *A. ordosica* plants followed the order of W + > W > W-. The soluble sugar and NSC contents of *A. ordosica* plants were non-difference under different precipitation treatments. These results suggest that the longer the precipitation interval and the lower the amount of precipitation, the lower the starch content of the whole *A. ordosica* plant.

At natural precipitation levels (W), the soluble sugar content of *A. ordosica* plants showed significant differences according to precipitation interval, being lower at T than at T++. At W- and W + levels, no significant difference was observed in the effect of precipitation intervals on soluble sugar content in the whole plant. At the W- level, the contents of starch and NSCs in *A. ordosica* plants were higher at T than those at T++, that is, the reduced whole-plant starch and NSC content was associated with longer precipitation interval. However, at the W and W + levels, longer precipitation interval did not result in significant changes in whole-plant starch content.

Precipitation amount had significant effects on the contents of soluble sugar and NSCs in whole *A. ordosica* plants (Table 1), whereas the precipitation interval had significant effects only on the starch content of the whole plants. The interaction between the two had significant effects on the soluble sugar, starch, and NSC contents in whole *A. ordosica* plants (P < 0.05).

### Analysis of NSC variability in different organs among precipitation change scenarios

**Fig. 2:**
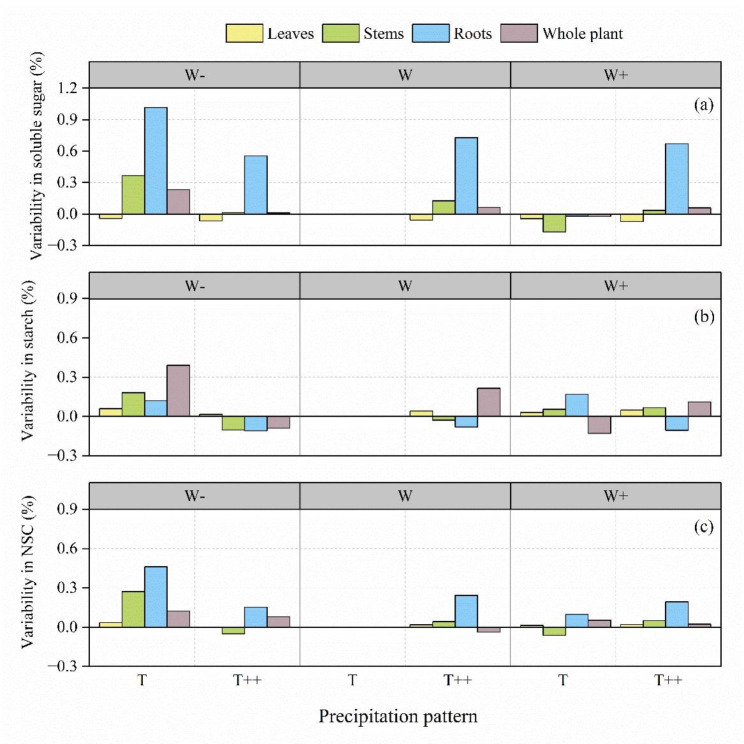
Effects of precipitation pattern (%) on non-structural carbohydrate (NSC) pools under different treatments compared with those in controls. Soluble sugar (**a**), starch (**b**), and NSC (**c**) in *Artemisia ordosica*

The variation coefficients with precipitation changing of soluble sugar content, starch content, and NSC content in each organ was observed as follows: roots > stems > leaves. This suggests that the regulation of non-structural carbon allocation in response to precipitation changing was primarily occurring in roots (Table 2). Compared to CK (WT treatment), W-T treatment had the greatest effects on NSCs and their components. Soluble sugar, starch, and NSC contents of *A. ordosica* significantly increased in stems and roots; the most significant increases were detected for soluble sugar and NSC contents of the roots, suggesting that *A. ordosica* plants allocated more carbon to roots in order to absorb more soil water in response to low precipitation amount. W + T treatment significantly reduced the soluble sugar content and increased the starch content in all organs, reduced the total NSC content in stems, and increased the NSC content in other organs, indicating that significant reduction in NSC content under drought stress occurred only in some organs. W-T++, WT++, and W + T + + treatments significantly reduced the root starch content but significantly increased the root soluble sugar and total NSC contents (Fig. 2a, b, c), indicating that in order to adapt to changes in rainfall patterns, the roots of *A. ordosica* plants responds to water stress through the interconversion between soluble sugar and starch. Overall, the root system of *A. ordosica* showed the greatest variability and was the main site of regulation for the distribution of carbohydrate components in the plant.

**Table 2:** Distribution characteristics of non-structural carbohydrates (NSC) and their components in various organs of *A. ordosica*

Index	Organs	Min. (%)	Max. (%)	Mean (%)	Standard deviation	Coefficient of variation (%)
Soluble sugar	Leaves	3.09	3.47	3.3061b	0.14130	4.27
	Stems	3.81	11.83	7.2878a	1.55772	21.37
	Roots	2.77	12.00	7.4767a	2.40887	32.22
Starch	Leaves	11.18	12.84	11.9339a	0.54809	4.59
	Stems	6.32	9.41	7.5272b	0.82715	10.99
	Roots	5.70	9.51	7.7017b	1.12121	14.56
NSC	Leaves	14.65	16.14	15.2400a	0.52400	3.44
	Stems	11.40	19.69	14.8156a	1.87283	12.64
	Roots	10.36	21.51	15.1778a	2.50626	16.51

Note: Different letters indicate significant difference at 0.05 level in different organs

### Effects of precipitation amount levels and intervals on the allocation of NSC components

Precipitation interval had a significant effect on the soluble sugar–starch ratio in the roots of *A. ordosica* (P < 0.05), whereas precipitation amount and its interaction with precipitation intervals showed no significant effect on the soluble sugar–starch ratio (P > 0.05) (Table 3).

**Table 3:** Two-way ANOVA on the effects of precipitation amount and precipitation interval on the soluble sugar–starch ratio of *Artemisia ordosica* seedlings (*F*-value)

Factor	Soluble sugar: starch			
	Leaves	Stems	Roots	Plant
W	0.743	2.719	1.556	3.708
T	1.615	1.080	12.883**	3.785
W×T	1.110	0.500	2.504	1.994

Note. ∗: P < 0.05, ∗∗: P < 0.01, ∗∗∗: P < 0.001. W: precipitation amount; T: precipitation interval

**Fig. 3:**
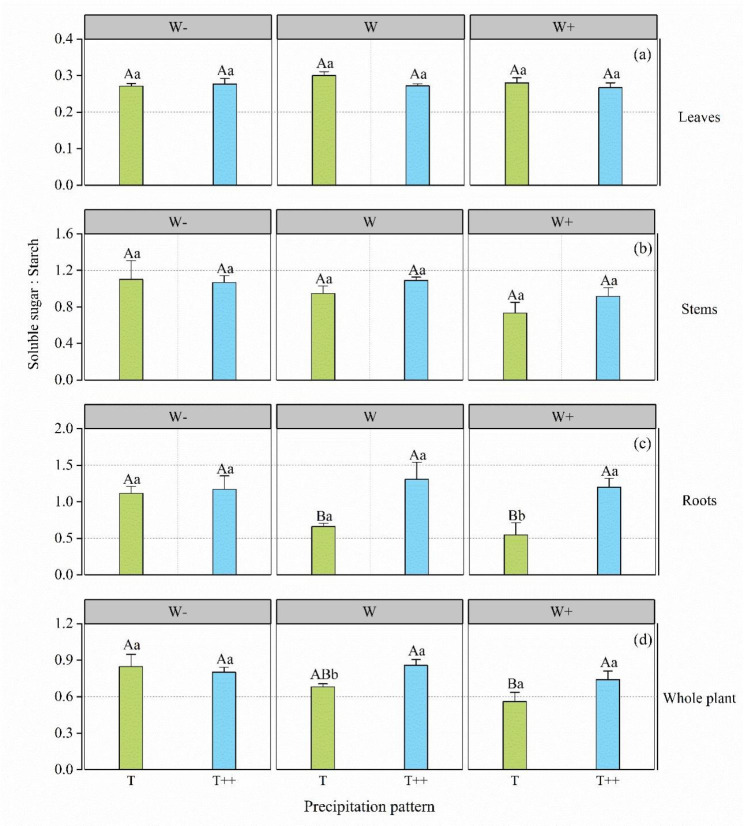
Effect of precipitation amount level and interval on the soluble sugar–starch ratio in *Artemisia ordosica*. Leaves (**a**), stems (**b**), roots (**c**), and whole plant (**d**). Error bars indicate ± 1 S.E. (*N* = 3). Note: Data in the figure are presented as the mean ± standard error (*n* = 3). Different uppercase letters show significant differences in precipitation amount under the same precipitation interval (P < 0.05); different lowercase letters indicate significant differences between the different precipitation intervals at the same precipitation level (P < 0.05)

At the T level, under different precipitation levels at T frequency, the soluble sugar–starch ratio in the leaves and stems of A. ordosica did not show significant differences and those in the roots and whole plants were significantly different in the order W- > W > W+ (Fig. 3c, d). At the T + + level, the soluble sugar–starch ratio in each organ and the whole plant of *A. ordosica* did not show significant differences under the different precipitation amount treatments. In other words, under the background of natural precipitation frequency, the decrease in precipitation amount increased the soluble sugar–starch ratio in roots and whole plants, whereas with longer precipitation interval, the decrease in precipitation had no effect on the soluble sugar–starch ratio in organs and whole plants.

At the W- level, the soluble sugar–starch ratio in each organ of *A. ordosica* showed non-difference at different precipitation intervals. At the W level, the soluble sugar–starch ratio in the whole *A. ordosica* plants was significantly different under different precipitation intervals, being higher at T + + than at T; however, at the W + level, the soluble sugar–starch ratio in each organ of *A. ordosica* only differed significantly in the root system, being higher at T + + than at T. In the context of natural precipitation, the longer the precipitation interval, the higher the soluble sugar–starch ratio of the whole *A. ordosica* plant. In the context of a 30% increase in precipitation amount, the longer the precipitation interval, the greater was the soluble sugar–starch ratio in the root system of *A. ordosica*. With prolonged precipitation interval, the soluble sugar–starch ratio in the roots of *A. ordosica* was greater than one, and the amount of soluble sugar in the roots of the plant was always greater than that of starch. However, the soluble sugar–starch ratio in whole *A. ordosica* plants was less than one (0.48–0.78; Fig. 3d), indicating that NSC in *A. ordosica* plants always contained more starch than soluble sugar. This finding indicates that *A. ordosica* predominantly stores NSC as starch.

## Discussion

Water resources affect the carbon assimilation yield of plants and thereby change the NSC content in plants [32]. Climate change has caused changes in the temporal patterns of precipitation, triggering droughts of varying intensity and frequency. However, in the face of drought events, plants usually store a certain amount of NSCs, which provide additional energy directed to water stress resistance [21]. A short period of drought inhibits plant photosynthesis, and the decrease in photosynthetic rate lags the growth rate, which leads to the accumulation of NSCs in plants [27, 28] (Fig. 1). However, a longer period of drought leads to more serious water stress, the balance between carbon uptake and carbon consumption of plants is disrupted [13], and the NSC content decreases or remains relatively stable when the NSC produced by photosynthesis cannot offset the NSC required for respiration, growth, and defense [33, 34] (Fig. 1). This suggests that the effect of precipitation amount on plant NSC component content is dependent on changes in precipitation intervals, consistent with our study results. Our findings indicate that the interaction between precipitation amount and interval had a significant effect on the soluble sugar, starch, and NSC content of whole *A. ordosica* plants (P < 0.05; Table 1). At natural precipitation frequency, the NSC and its components increased in whole *A. ordosica* plants with decreasing precipitation amount, and when the precipitation interval was extended to 15 days, the starch content decreased and the NSC content remained relatively stable with decreasing precipitation in whole *A. ordosica* plants. This may be because, with natural precipitation frequency, a decrease in precipitation leads to insufficient soil water supply, which inhibits the normal physiological and metabolic activities of *A. ordosica*, and the accumulation of synthetic photosynthates in the plant body leads to an increase in NSC content. However, at longer precipitation intervals, *A. ordosica* can regulate photosynthesis and respiration through its homeostatic mechanism to maintain the relative stability of NSCs [8, 22, 23]. In addition, our results indicated that when precipitation amount was reduced by 30% and the precipitation interval was extended, the NSC content of *A. ordosica* plants was reduced only in the stems, and no significant changes were observed in other organs (Fig. 1). Because carbon stored in plants cannot be fully utilized, or because the carbon available to the plant is limited in arid environments, carbon depletion does not occur even when plants die [16, 33], and a decrease in NSC content does not occur in all organs [22, 35, 36], which also verifies our results. The distribution pattern of NSC components is a result of the synergistic action of physiological processes, reflecting the adaptation mechanisms of plants to environmental factors [9, 13], which are closely related to plant survival strategies [37]. The conversion of NSCs between carbon utilization and storage via the soluble sugar and starch is an effective mechanism for plant stress resistance [38]. When soluble sugar accumulates to a high level, it can be converted and stored in the form of starch; conversely, when soluble sugar content is low, starch is decomposed and converted into sugar to maintain normal physiological activities [39]. Many studies have shown that when plants are subjected to drought stress, they often maintain or even increase soluble sugars at the cost of starch consumption, mainly because soluble sugars play a role in regulating osmotic pressure and maintaining cell turgor pressure under drought conditions [16, 17, 25, 40, 41]. Our study found that precipitation interval had a significant effect on the soluble sugar–starch ratio in the roots of *A. ordosica* (P < 0.05). Compared with the control (WT), extended precipitation interval (W-T++, WT++, W + T++) resulted in an increase in the soluble sugar content of the roots of *A. ordosica* together with a decrease in starch content, and the soluble sugar–starch ratio was greater than 1 (Figs. 2 and 3). This suggests that, mutual conversion of soluble sugars to starch is a mechanism of *A. ordosica* for adaptation to the changing external environment initiated by water stress due to extended precipitation interval. By hydrolyzing root starch, *A. ordosica* supplements the plant’s demand for soluble sugars, increases the concentration of osmotic regulators, maintains the water balance of cells, and ensures the root system’s ability to absorb water and nutrients [14, 18], which is an adaptive strategy enabling *A. ordosica* to save energy and maintain normal physiological metabolism. Meanwhile, the root system was found to be the major part of the regulation mechanism of carbohydrate distribution in *A. ordosica*.

At the same time, the allocation of NSCs among different plant organs can buffer the different synchronisms of carbon supply and demand among different organs under stress and maintain plant osmoregulation, growth, and development [38]. The allocation of NSCs to different organs depends on the ability (or competition) of each organ to use NSCs. Generally, plants follow the “preferential allocation principle” prioritizing carbon supply to the most important organs [42] and growing organs receiving NSCs first [43, 44]. Our study found that, compared with the control treatment, under natural normal precipitation frequency and reduced precipitation amount, the NSC content in the stems and roots of *A. ordosica* increased dramatically, especially soluble sugar in roots. This suggests that under drought stress *A. ordosica* allocates more carbon to underground parts to maintain the survival of the root, which was consistent with the hypothesis of optimal allocation [45, 46]. Previous studies have shown that in conditions of drought stress, plant carbon input is always insufficient to meet plant carbon demands (such as respiration, metabolism, defense, and osmoregulation functions). Therefore, in order to maintain survival and improve nutrient utilization efficiency, NSCs are gradually accumulated in the stems and then rapidly transferred to roots [47, 48]. Simultaneously, part of the starch in the root system is hydrolyzed into soluble sugars, which regulates the osmotic potential while maintaining leaf cell metabolic activity [17, 49]. After drought period, tissue and organ reconstruction are performed under optimal environmental conditions [50, 51], which is an inter-organ coordinated adaptation strategy of *A. ordosica* to cope with changes in the drought environment.

## Conclusions

The interaction between precipitation amount and interval had significant effects on the content of NSCs and their components in *A. ordosica* plants. The effect of precipitation amount on the content of NSCs and their components in *A. ordosica* depends on the variability in precipitation interval. At natural precipitation frequency, with a decrease in precipitation amount, the contents of soluble sugar in roots and starch in stems of *A. ordosica* gradually increased. In contrast, under prolonged precipitation interval, the starch content of the whole plant decreased as precipitation decreased.

The interconversion of NSC components and coordinated allocation between organs are adaptive strategies for *A. ordosica* to cope with changes in precipitation. Lower precipitation amount and longer precipitation interval led to an increase in the soluble sugar and starch fractions of whole *A. ordosica* plant, and NSC prefer to be allocated more in the roots systems. These results suggest that root system was the main regulatory site of carbohydrate distribution in *A. ordosica.*

This study provided new ideas for the adaptation mechanisms of plants in deserts to global environmental changes, and offered a new insight into the vegetation structure in desert regions under global change scenarios.

### Electronic supplementary material

Below is the link to the electronic supplementary material.

Supplementary Material 1

## Data Availability

Data are made available as supplementary material.
